# Supplementary material for: OSCA: a tool for omic-data-based complex trait analysis
Source: Genome Biol. 2019 May 28;20:107. doi: 10.1186/s13059-019-1718-z (PMC6537380; doi:10.1186/s13059-019-1718-z)
Supplement: Supplementary file 2 — Review history. (DOCX 21 kb) [file 13059_2019_1718_MOESM2_ESM.docx]

Review History

**First round of review**

**Comments to author and authors’ point-by-point responses to the reviewers’ comments:**

**Reviewer 1**

Zhang et al. presents a novel method MOMENT for performing association studies of complex traits based on omics data. MOMENT is based on mixed linear model that incorporates multiple random-effect components to account for hidden confounders. Through simulation studies and a real example of epigenome-wide association study, they show MOMENT achieves a lower false positive rate and is more robust than existing methods.

MOMENT has been implemented into a software package OSCA. The paper structure is clear and easy to follow. The conclusion is supported by the presented data and results. The tool will have a wide range of applications in many omics studies.

I have a few comments outlined below:

1. It's easy to follow the logic using the linear mixed model to control for the covariance among all cpg sites. It's mathematically straightforward, but it could be computationally difficult. To my understanding, authors estimate the covariance matrix first and fix it when updating other parameters for the test cpg and covariates, there is a m x m matrix calculation for each cpg. If the platform is 450k chip, it would be a 450k x 450k matrix calculation repeating for 450k time to parse through the whole methylome. Although the software was implemented in C++, which should be fast for the matrix calculation, it is unclear if approximate approach has been used when m is large. Can the authors show the time (for both exact and approximate approach) used in your real methylome-wide studies with server/computer information? It would be also show some comparisons of computational complexity among all tested methods should be discussed.

*Response:* *The MOA/MOMENT analysis requires the computation of an n x n matrix (where n is the number of individuals rather than the number of probes), referred to as the omic-data-based relationship matrix (ORM) in our manuscript (pages 5 and 16). For MOA, the ORM does not need to be computed repeatedly because all the probes are included in the random-effect term when testing for a target probe. The most time-consuming step in MOA is the computation of V^-1^(where V is the phenotype variance and covariance matrix; see pages 5, 6 and 16) which in principle needs to be done repeatedly in each REML iteration for each target probe. This issue has been discussed in the manuscript (page 6). The solution is to use an approximate approach that only requires to compute V^-1^once (page 6). We have shown an extremely high correlation in -log10(p-value) between the approximate and exact MOA approaches (Pearson correlation = 0.9998 for causal probes and ~1.0 for null probes; Supplementary Figures 1a and 1b). With regard to MOMENT, there are two ORMs (corresponding to two random-effect components) and probes in close physical proximity of the target probe need to be removed from the ORMs. Note that the computational time to adjust the ORMs (because of removal of some of the probes) is minimal. We have also implemented the approximate approach in MOMENT with a small modification that the variance components are re-estimated (under the null) when one or more probes are removed from the first ORM in case that the variance captured by some of the probes in the first ORM are large. The test-statistics from approximate MOMENT are also extremely highly correlated with those from exact MOMENT (Pearson correlation = 0.9993 for causal probes and 0.9981 for null probes; Supplementary Figures 1c and 1d). We have commented on this in the revised manuscript (page 7). The computing time of all the methods have been quantified in the LBC data using the simulated phenotypes (Supplementary Table 3).*

*In the revised manuscript, we have shown the computational complexity of the methods as well as their computing time in application to a real phenotype in the LBC (Supplementary Table 3).*

2. The framework is based on linear mixed model for continuous traits. The authors mentioned that this method could be also applicable to case-control trait. Do you mean you still keep the sigma_e for random error in the formulas, and just assume case-control trait follows normal (e.g. use probit model)? If so, it's not ideal. A more general framework, GLMM, could be used to replace LMM to achieve this. Certainly, the computation burden would be much elevated. More discussion can be added it because binary trait is widely existing in many applications.

*Response: We thank the reviewer for raising this point. The use of linear models for binary traits have been justified in previous studies (Lee et al. 2011 AJHG; Pirinen et al. 2013 Ann Appl Stat) and mixed linear models have been widely applied in the analysis of binary phenotypes in genome-wide association studies (Kang et al. 2010 Nat Genet; Zhou and Stephens 2012 Nat Genet; Listgarten et al. 2012 Nat Methods; Yang et al. 2014 Nat Genet; Liu et al. 2015 Nat Genet). We have included this in our manuscript (page 12). In the revised manuscript, we have shown by simulation that both MOMENT and MOA performed well when applied to case-control phenotypes regardless whether cases were oversampled (Supplementary Figures 15 and 16; page 10).*

*We agree with the reviewer that direct application of linear model approaches to case-control phenotypes is not ideal. If the underlying model is causal (i.e., omic measures have causal effects on the traits), a more appropriate analysis is to use a link function (e.g., a probit or logit model) that connects the case-control status to a latent continuous trait, as in the methods developed recently for the analysis of case-control data in GWAS (Weissbrod et al. 2015 Nat Methods; Hayeck et al. 2015 AJHG; Chen et al. 2016 AJHG; Zhou et al. 2018 Nat Genet). Since OSCA is an ongoing software development project, some of these methods can be incorporated in the MOMENT framework in the future. We have commented on this in the revised manuscript (pages 14 and 15).*

3. The target of the method is for any omics data. However, only methylation data were tested. It would be good to add one more example of transcriptomic-wide association study to illustrate the wide application of this tool. At least, some discussion should be added.

*Response:* *We thank the reviewer for this suggestion. We have tested the methods by simulation based on a real gene expression data set (19,648 gene expression probes on 1,219 individuals from the San Antonio Family Heart Study (SAFHS)) under simulation scenario 1 (i.e., quantitative phenotypes simulated based on the expression levels of 100 randomly selected causal probes) (page 19). Both MOMENT and MOA performed similarly (in comparison to the other MWAS methods) as in the simulations based on DNAm data (Supplementary Figure 17). We have commented on this in the revised manuscript (page 10).*

4. Minor: In the abstract, omic-data-based quantitative trait locus (xQTL) is a bit misleading. xQTL often refers to eQTL (RNA ~ snp) or meQTL (Methylation ~ snp) while here quantitative trait refers to clinical phenotype such as lung function or BMI.

*Response: This is actually what we meant. In the previous version of the manuscript, we stated in the Abstract that MOMENT is implemented in OSCA together with a number of other implementations including xQTL analysis.*

*“MOMENT has been implemented in a versatile software package (called OSCA)* ***together with a number of other implementations*** *for omic-data-based analysis including the estimation of variance in a trait captured by all measures of multiple omic profiles, omic-data-based quantitative trait locus (xQTL) analysis.”*

*Note that this statement has been removed from the revised manuscript according to the*

*editorial instruction (i.e., <100 words in the Abstract).*

**Reviewer 2**

This article describes a mixed linear model-based method ('MOMENT') that tests association between traits and DNA methylation status at individual probes while accounting for non-specified potential confounders (e.g. cell-type composition of assayed tissue). To quantify the false positive rate (FPR) and power of MOMENT in comparison with other methods, the authors perform simulation analyses using DNA methylation and known cell-type composition data from blood samples from the Lothian birth cohort. They show a surprisingly high FPR due to confounding in existing methods, which is controlled by MOMENT. This is further demonstrated using data on body mass index, height, lung function and walking speed from the cohort. The manuscript is well-written and the software is likely to be very useful to investigators conducting methylation-wide association studies. I have the following comments:

1. Although the title is 'OSCA: a tool for omic-based complex trait analysis', the manuscript focuses on MOMENT, which is only one component of the OSCA software package. I therefore think a title around MOMENT would be more appropriate.

*Response: We aim in our paper to introduce a software tool for complex trait analyses using multi-omic data. The reviewer is correct that the manuscript has focused heavily on MOMENT and MOA just because they are novel methods which need to be tested thoroughly by simulations, robustness tests, real data analyses. The software tool does include other implementations such as OREML (to estimate the proportion of variance captured by all measures of one or multiple omic profiles), BLUP (to predict a phenotype based on one or multiple omic profiles), PCA analysis, xQTL analysis (to detect associations of genetic variants with omic measures), and meta-analysis of the xQTL data. As stated in the manuscript, OSCA is an ongoing software development project.*

*More functionalities and methods will be included in the software package, such as LASSO and elastic net for phenotype prediction and generalised mixed linear models for association analysis of case-control phenotypes. Moreover, we have deposited the manuscript in bioRxiv and the preprint has been cited in multiple manuscripts in the pipeline. We therefore think that it is better to keep the current title unchanged.*

2. It is stated (page 4, lines 83-85) that the methods are in principle applicable to other types of omic data including gene expression, DNAm (note that this shouldn't be repeated in this sentence), histone modification and protein abundance. However, I'm not entirely sure that features of these data types would make the method more generally applicable. For example, the method excludes probes in close proximity to the target from the random effect term because they tend to show correlated methylation due to the same regulatory mechanism. This is unlikely to be the case for gene expression microarray (where each gene is typically tagged by a single probe) or RNA-Seq data. I therefore think that some evidence to support this claim should be provided, or the method be more specifically described as a tool for DNA methylation-based studies.

*Response:* *We thank the reviewer for raising this point and have revised the text according to the reviewer’s comment (page 4).*

*We have tested the methods by simulation based on gene expression data from the San Antonio Family Heart study (SAFHS) under simulation scenario 1 (i.e., quantitative phenotypes simulated based on the expression levels of 100 randomly selected causal probes). Both MOMENT and MOA performed similarly (in comparison to the other MWAS methods) as in the simulations based on DNAm data. We have included this result in the revised manuscript (page 10).*

*The reviewer is correct that the correlation structure of gene expression probes is different from that of DNAm probes. We have quantified the decay of correlation between a pair of gene expression probes as a function of their physical distance (Supplementary Figure 30), which suggests that 100Kbp is an appropriate MOMENT window size for gene expression data although the MWAS results remained almost unchanged when the window size was varied from 50Kbp to 1Mbp (Supplementary Figure 31). We have commented on this in the revised manuscript (page 14).*
